# The German Lipoprotein Apheresis Registry (GLAR) – almost 5 years on

**DOI:** 10.1007/s11789-017-0089-9

**Published:** 2017-02-23

**Authors:** V. J. J. Schettler, C. L. Neumann, C. Peter, T. Zimmermann, U. Julius, E. Roeseler, F. Heigl, P. Grützmacher, H. Blume, A. Vogt, Scientific Board of GLAR for the German Apheresis Working Group

**Affiliations:** 1 Center of Nephrology Göttingen GbR, An der Lutter 24, 37075 Göttingen, Germany; 2 BRAVE e Benefit for Research on Arterial Hypertension, Dyslipidemia and Vascular Risk and Education e. V., Göttingen, Germany; 3 BioArtProducts GmbH (B.A.P.), Rostock, Germany; 4 Rostock Group (EXIM), Fraunhofer Institute for Cell Therapy and Immunology IZI, Rostock, Germany; 5 Department of Medicine III, University Hospital Carl Gustav Carus, Technische Universität Dresden, Dresden, Germany; 6 Center for Nephrology, Hypertension, and Metabolic Diseases, Hannover, Germany; 7 Medical Care Centre Kempten-Allgäu, Kempten, Germany; 8 Department of Medicine II for Nephrology, Hypertension and Vascular Risks, AGAPLESION Markus Hospital, Frankfurt, Germany; 9 Scientific Institute for Nephrology (WiNe), Düsseldorf, Germany; 10 Medizinische Klinik und Poliklinik 4, Universität München, Munich, Germany

**Keywords:** Lipoprotein apheresis, Lipoprotein apheresis registry, Coronary heart disease, Risk reduction, Prevention

## Abstract

**Background:**

Since 2005 an interdisciplinary German apheresis working group has been established by members of both German Societies of Nephrology and of Lipidologists and completed the data set for the registry according to the current guidelines and the German indication guideline for apheresis in 2009. In 2011 the German Lipoprotein Apheresis Registry (GLAR) was launched and data are available over nearly 5 years now.

**Methods and results:**

During the time period 2012–2016, 71 German apheresis centers collected retrospective and prospective observational data of 1435 patients undergoing lipoprotein apheresis (LA) treatment of high LDL-C levels and/or high Lp (a) levels suffering from cardiovascular disease (CVD) or progressive CVD. A total of 15,527 completely documented LA treatments were entered into the database. All patients treated by LA showed a median LDL-C reduction rate of 67.5%, and a median Lp (a) reduction rate of 71.1%. Analog to the Pro(a)LiFe pattern, patient data were analyzed to the incidence rate of coronary events (MACE) 1 and 2 years before the beginning of LA treatment (y-2 and y‑1) and prospectively two years on LA treatment (y + 1 and y + 2). During two years of LA treatment a MACE reduction of 78% was observed. In the years considered, side effects of LA treatment were low (5.9%) and mainly comprised puncture problems.

**Conclusions:**

The data generated by the GLAR shows that LA lowers the incidence rate of cardiovascular events in patients with high LDL-C and/or high Lp (a) levels, progressive CVD, and maximally tolerated lipid lowering medication. In addition, LA treatments were found to be safe with a low rate of side effects.

## Introduction

Up to the early 80s of the last century, no adequate lipid lowering therapy was available for patients suffering from progressive cardiovascular diseases induced by severe hyperlipidemia, mainly high LDL-cholesterol (LDL-C) levels or Lp (a)-hyperlipoproteinemia (Lp (a)-HLP (a)) [1]. Since then, lipoprotein apheresis (LA) has been developed to treat a subset of these patients [2]. During the 80s and 90s many trials with respect to therapy by different statins were performed. They showed that in patients suffering from severe hypercholesterolemia a decrease of LDL-C is associated with a decrease of cardiovascular events in both primary and secondary prevention [3]. However, for LA patients with cardiovascular disease (CVD) suffering from high LDL-C and/or Lp (a) levels only a few studies are available demonstrating an additional LA effect on cardiovascular risk reduction [4–6]. In the 2000s the Federal Joint Committee (G-BA), a paramount decision-making body of the German healthcare system, called for a reassessment of the approval of chronic LA therapy for regular reimbursement. At the same time an interdisciplinary German apheresis working group was established by members of both German Societies of Nephrology and Lipidologists, who developed an indication for LA with respect to current cardiovascular guidelines and scientific knowledge and initiated the establishment of the German Lipoprotein Apheresis Registry (GLAR) [7, 8]. GLAR was launched in 2011 [9, 10] and data collected over almost 5 years are available.

## Materials and methods

### Access to the German Lipoprotein Apheresis Registry (GLAR)

Before an apheresis center is eligible to enter patient data into the database, an application has to be filed with the Stiftung für Nephrologie e. V. (Foundation for Nephrology, founded by Verband Deutsche Nierenzentren, Duesseldorf, Germany). A cooperation agreement between the above-named foundation and the respective apheresis center is then concluded. Amongst other things this document clarifies data security aspects and reporting duties of the responsible body (foundation). After signing the agreement, the apheresis center is assigned a center key in form of a unique number. Only the foundation has knowledge of the associated center name, but not the technical service provider, BioArtProducts (B.A.P., Rostock, Germany). The foundation announces new participating centers to the service provider in an anonymized form providing the center key. Subsequently, B.A.P. creates login credentials for the online platform (https://apherese-register.org) and provides these to the foundation who in turn forwards them to the participating center. Only then is the center enabled to participate in the GLAR via the online platform.

Current German data protection laws require all participating patients of the respective apheresis centers to provide written informed consent to participation in the GLAR prior to any pseudonymized data being entered into the registry. The consent declarations are archived by the respective center. Analog to the indexing of centers, all patients are assigned a key in form of a unique number issued by their treating apheresis center. This ensures that only the treating center is able to link patient identity to patient data via the patient key.

The foundation as the responsible body has assigned the Institute for Nephrology (WiNe) with the operation of the registry.

### Data base and data quality

The database is developed in MySQL (Cupertino, California, USA) with GlassFish (Oracle Corporation, Redwood City, California) as server technology running on an Ubuntu system (Linux operating system, Canonical Ltd, UK). High data quality is supported by programmed range checks, validity checks, and consistency checks of the data entered.

### Data entry

Once access to GLAR is initiated, every patient’s data is handled like an electronic medical file containing e. g. medical history, relevant family history, diagnoses, medications, laboratory results, and side effects of apheresis treatments.

The scientific board of the GLAR requests quarterly documentation of a representative LA treatment including all lipid parameters before and after the treatment procedure and progress of any vascular diseases, e. g. coronary heart disease, stroke, as well as side effects associated with the LA treatment. Changes in lipid-lowering therapy strategy and reasons for patients to drop out of the registry are documented promptly.

### Data queries and evaluation

The latest database query for this publication was performed in November 2016. All data were collected in the time period 2012–2016 (until October 2016) and analyzed using the statistics software SigmaStat (SigmaStat 4.0; Systat Software Inc., San Jose, California, USA).

Additionally, patient data were analyzed with respect to incidence rates of major coronary events (MACE) and major non-coronary events (MANCE) 1 and 2 years before (y-2 and y‑1) and prospectively 1 and 2 years after initiation of LA treatment (y + 1 and y + 2). This current data pattern gives the optimal sample size for statistical evaluation. MACE was defined as an outcome parameter, i. e., cardiovascular death, nonfatal myocardial infarction, coronary bypass surgery, percutaneous coronary intervention, or stent, whereas MANCE was determined as non-cardiovascular events i. e. stroke, carotid percutaneous transluminal angioplasty or carotid surgery or peripheral vascular events (peripheral vascular event of lower extremities or renal arteries = percutaneous transluminal angioplasty, stent, bypass surgery, amputation), or venous thrombotic events = deep venous thrombosis or pulmonary embolism.

## Results

Data of more than 19,800 LA treatments of 1435 patients were entered into GLAR, mostly 1‑2 treatments per quarter. With more than 78% of the final documentation forms being complete 15,527 LA treatments were finally analyzed. All required data are available for each patient as the design of the entry form requires a complete data collection.

From 2012 to 2016, 71 German apheresis centers collected retrospective and prospective observational data of 1435 patients undergoing LA to treat high LDL-C levels and/or high Lp (a) levels, who were suffering from progressive cardiovascular disease (CVD). Until 2015 the majority of patients were female (838 females vs 441 males) with most of them being 60 years or older (Table 1).

**Table 1:** Age and sex distribution in the GLAR database (data interrogation until 2015)

Age	Males	Females	Total
Under 18			
18–30	3	4	*7*
30–39	6	21	*27*
40–49	23	88	*111*
50–59	105	231	*336*
60–69	127	256	*383*
70 and older	177	238	*415*
*Total*	*441*	*838*	*1279*
Oldest patient			90 years old
Youngest patient			20 years old

To qualify for reimbursement of LA therapy the German guideline for LA treatment requests Lp (a) levels above 60 mg/dl (120 nmol/l) and normal LDL levels (mainly <100 mg/dl) in addition to clinical features. Therefore, we analyzed the data for those levels.

Until 2015 with respect to the patient population of the GLAR, 50.8% (*n* = 544) of the included patients had Lp (a) levels ≥60 mg/dl (≥120 nmol/l), 23.3% (*n* = 325) LDL‑C levels ≥100 mg/dl (≥2.6 mmol/l), and in 51.5% (*n* = 551) both levels (Lp(a) levels ≥30 mg/dl (≥70 nmol/l); LDL‑C levels ≥100 mg/dl (≥2.6 mmol/l)) were elevated despite maximally tolerated cholesterol lowering drug therapy.

All patients were treated by LA with a median acute LDL-C reduction rate of 68.6%, and median acute Lp (a) reduction rate of 70.4%. The detailed median annual reduction rates of LDL-C levels were 68.5% (*n* = 449) in 2012, 68.7% (*n* = 695) in 2013, 68.7% (*n* = 916) in 2014, and 67.5% (*n* = 1070) in 2015. The median annual reduction rates of Lp (a) levels were 68.8% (*n* = 449) in 2012, 69.6% (*n* = 695) in 2013, 71.3% (*n* = 916) in 2014, and 71.1% (*n* = 1070) in 2015.

The rate of LA treatment side effects remained low (ca. 5%) (mainly puncture problems) during the observed years (Table 2).

**Table 2:** Top 5 of side effects documented in the GLAR between 2012 and 2015

Year	2012	2013	2014	2015
Treatments per year	1674	3181	5007	5305
Puncture problems	14 (0.84%)	51 (1.6%)	99 (1.98%)	150 (2.83%)
Hypotension	17 (1.02%)	26 (1.28%)	64 (1.28%)	58 (1.09%)
Unclassified effects	10 (0.6%)	15 (0.47%)	33 (0.66%)	48 (0.9%)
Technical failures	7 (0.42%)	6 (0.19%)	21 (0.42%)	35 (0.66%)
Dizziness	1 (0.06%)	8 (0.25%)	23 (0.46%)	20 (0.38%)
Total	49 (3.0%)	106 (3.3%)	240 (4.8%)	311 (5.9%)

**Fig. 1:**
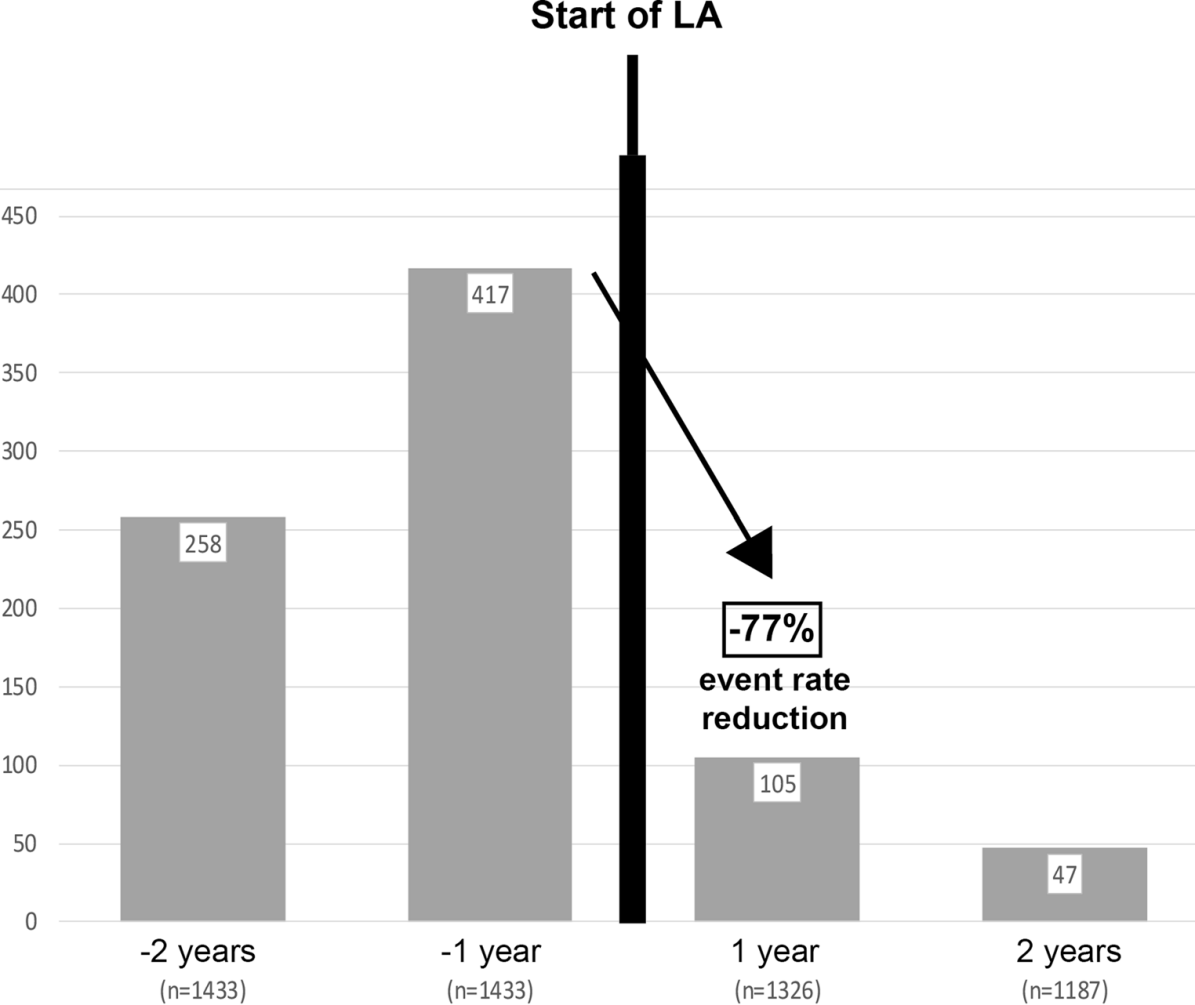
Major Coronary Events (MACE) 1 and 2 years before (y-2 and y‑1) and two years on LA treatment (y + 1 and y + 2). GLAR data interrogation 2012 and 2013, in which these event patterns can be found

**Fig. 2:**
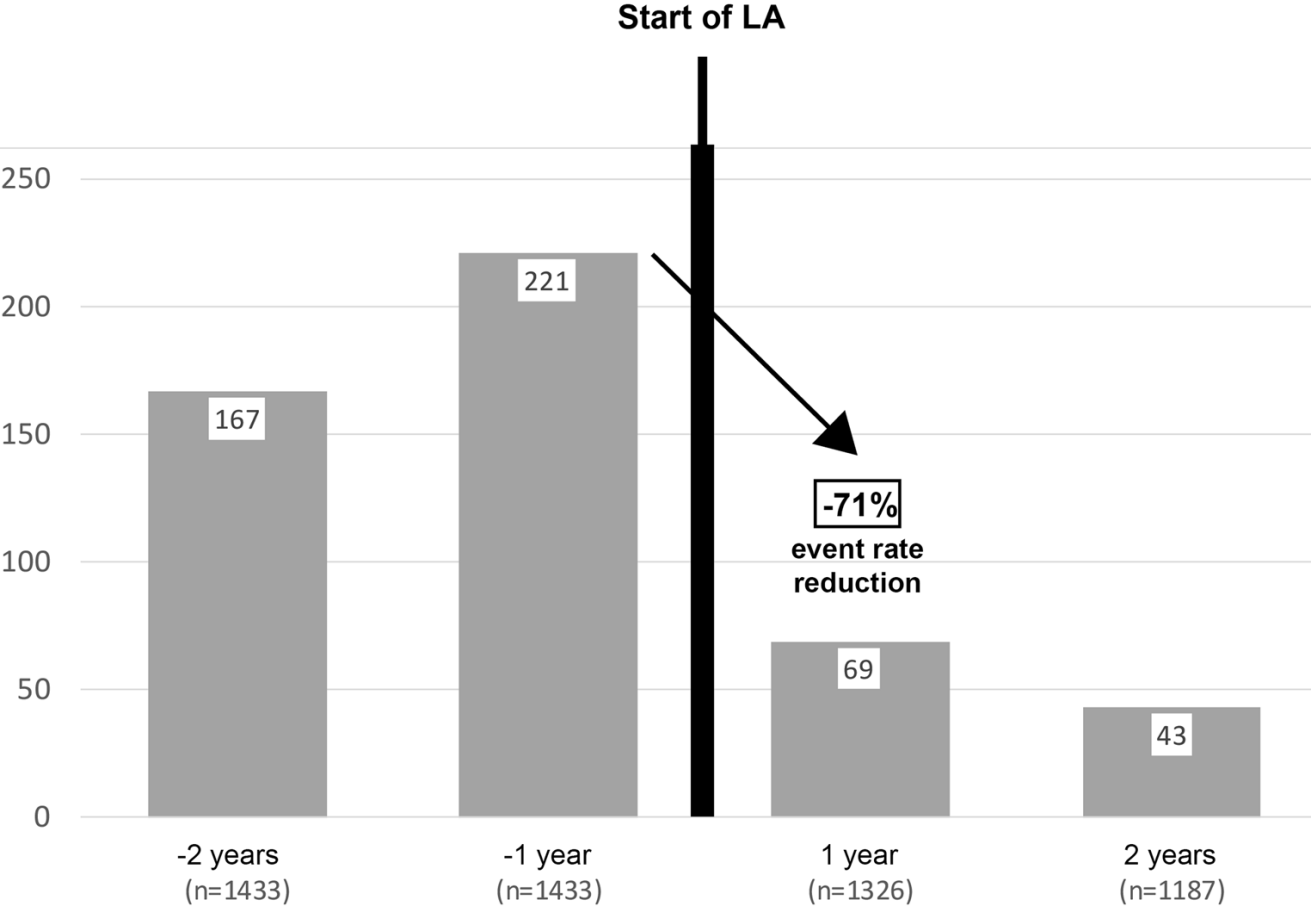
Major Non-Coronary Events (MANCE) reduction rate 1 and 2 years before (y-2 and y‑1) and two years on LA treatment (y + 1 and y + 2). GLAR data interrogation 2012 and 2013, in which these event patterns can be found

Analog to the pattern of the Pro(a)LiFe study, patient data were analyzed with respect to incidence rates of major coronary events (MACE) 1 and 2 years before (y-2 and y‑1) and prospectively two years on LA treatment (y + 1 and y + 2). Patients with available data according to the query pattern (data points for y‑2, y‑1 and y + 1, y + 2 in the period 2012–2016) were found to have a reduction in MACE of 77% (Fig. 1). The same query scheme was applied for major non-coronary events (MANCE) (Fig. 2). An average reduction rate of MANCE of 71% was observed.

For both, MACE and MANCE, the above detailed data were available for varying numbers of patients over the years. Therefore, events are presented as median over the entire time interval 2012–2015 (Figs. 1 and 2).

## Discussion

In 2000s the Federal Joint Committee (G-BA) called for the collection of clinical outcome data from patients undergoing regular LA therapy. At the same time an apheresis working group was founded in order to create an LA registry (GLAR). With respect to other international and national apheresis registries, this is the first registry to record such a large amount of LA treatments and LA patients [11, 12]. After encouraging preliminary results, we now report on an analysis of data collected over almost 5 years [9].

The GLAR represents a multi-center acquisition of data of more than 15,000 single LA treatments, which to the best knowledge of the authors is the highest number of cases collected world-wide. The data prove a very high quality of treatment with respect to the reduction of LDL-C and Lp (a) levels and underline the potency of LA as therapeutic means for patients suffering from, in parts multiple, cardiovascular events.

This result was obtained with a low number of documented non serious side effects like hypotension which is a potential side effect of extracorporeal therapies in general and not specific to LA treatment. The most common side effects not related to the procedure were issues with punctures. The light increase of puncture problems may be explained by the increase of new patients documented in GLAR. In the last four years very few drop-outs were observed (on average 30 patients per year; 4.3% of all patients) from GLAR, mainly patient-initiated (41%) or death (30%).

Following the pattern of the Pro(a)LiFe-study, an additional evaluation of coronary events (MACE) and non-coronary events (MANCE) two (y-2) and one year (y-1) prior to LA treatment as well as two years on LA treatment (y + 1 and y + 2) was performed. It is noteworthy that the impressive reductions are consistent with the data of the Pro(a)LiFe-study. Reduction rates of MACE (−72 and −78%, respectively), MANCE (−71 and −75.9%, respectively) were striking already in the first year after the onset of LA [5]. We assume that the events documented here are potentially comparable as well to data on survival rates reported by the Pro(a)LiFe-study [5, 13]. However, it has to be kept in mind that the reported data are based on entries in the GLAR, only. As the registry is based on voluntary participation, limitations to documentation quality, completeness of data, and regularity of data recordings do exist. Additionally, the number of participating apheresis centers remained nearly constant in the last two years. This might be beneficial for the data quality due to the more experienced staff of those centers. Nonetheless, more efforts should be done to increase the number of participating apheresis centers to support the concept of GLAR as a representative LA database of Germany. Since the term of GLAR is currently extended to 2019 at least, throughout the next years data of LA patients covering longer intervals will become available. As in the follow-up of the recently published Pro(a)LiFe-study, evaluations of MACE and MANCE rates of patients undergoing LA treatment for 3, 4, or 5 years will be possible [14].

Moreover, the new PCSK9-inhibitors show promising effects on the lipid status of patients currently receiving LA treatment. In the currently documented apheresis treatments of patients with elevated LDL-C levels these substances so far have not shown to have an effect on participation rates and/or discontinuation of apheresis therapy [15]. This is likely to change in 2016/2017 as in the meantime two PCSK9-inhibitors have been approved by the BfArM.

Due to the high LDL-C level lowering potential of PCSK9-inhibitors, a certain number of patients are expected to do without LA treatment. The recently published ODYSSEY ESCAPE trial showed a discontinuation in 63.4% of patients on alirocumab who were previously undergoing regular LA [15]. However, according to the study design the reason for stopping LA was not a satisfactory LDL-C level but a general reduction of 30% of the pre-LA LDL-C level. 36.6% of patients had to continue LA procedure. In addition, in some patients with discontinued LA treatment LDL-C increased again at levels, which were higher than the requested target levels for LDL-C by guidelines. Since clinical decision-making is based on target levels GLAR will give insights of the effects of PCSK9-inhibitors in real life. To this point, no end-point studies exist which would allow to justify the broad use of PCSK9-inhibitor therapy in patients currently undergoing LA treatment [16] and suffering from, often multiple, cardiovascular events. Furthermore, it has to be emphasized that for patients with elevated Lp (a) levels (>60 mg/dl; >120 nmol/l) there is to date no alternative to a continued regular LA treatment [17]. In other patients, the new and promising PCSK9-inhibitor therapy may allow to reduce LDL-C concentration to a target value <70 mg/dl (<1.8 mmol/l) for the very first time [17], either as single therapy or in combination with statin medication and LA treatment. An additional focus of the GLAR for the coming years will be to observe if PCSK9-inhibitors will allow stopping or even preventing a progress of cardiovascular disease either as single therapy or in combination with other therapies. The GLAR will play an important role for generating evidence with respect to novel pharmacological developments, such as PCSK9-inhibitors, and their therapeutic potential, as well as their different capabilities compared to LA treatment.
